# Comparison of a quantitative real-time polymerase chain reaction (qPCR) with conventional PCR, bacterial culture and ELISA for detection of *Mycobacterium avium* subsp. *paratuberculosis* infection in sheep showing pathology of Johne’s disease

**DOI:** 10.1186/2193-1801-2-45

**Published:** 2013-02-11

**Authors:** Ganesh G Sonawane, Bhupendra N Tripathi

**Affiliations:** 1 Indian Veterinary Research Institute, Regional Station Palampur, Kangra, (HP-176062) India; 2 CCS National Institute of Animal Health, Baghpat-UP, India

**Keywords:** Bacterial culture, ELISA, *Mycobacterium avium* subsp*. paratuberculosis*, PCR, qPCR, Sheep

## Abstract

A quantitative real-time PCR (qPCR) assay employing IS*900* gene specific primers of *Mycobacterium avium* subsp. *parartuberculosis* (MAP) was compared with conventional PCR, bacterial culture and enzyme-linked immunosorbent assay in 38 sheep showing granulomatous enteritis and lymphadenitis with and without demonstration of acid-fast bacilli (AFB). The lesions were classified as multibacillary (MB) (n = 23), which had diffuse granulomatous lesions with abundant AFB, and paucibacillary (PB) (n = 15), which had focal or multifocal granulomatous lesions with few or no AFB. In the multibacillary group (MB), IS*900* PCR detected 19 (82.6%), and qPCR detected all 23 (100%) sheep positive for MAP in the intestine and lymph node tissues. In the paucibacillary group (PB), IS*900* PCR detected 2 (13.3%), and qPCR detected all 15 (100%) sheep positive for MAP in tissues. When results of both groups were taken together, IS*900* PCR detected 21(55.2%), and qPCR detected all 38 (100%) animals positive for MAP genome either in the intestine or lymph node tissues. On Herrold egg yolk medium, tissues of 14 (60.9%) MB and 5 (33.3%) PB sheep were found to be positive for MAP. Out of 27 sheep (PB = 8, MB = 19) tested by an ELISA, 21 (77.7%) were found to be positive for MAP antibody, of which 25% (2/8) and 100% (19/19) sheep were from PB and MB sheep, respectively. Based on the results of the present study, it was concluded that qPCR was a highly sensitive test in comparison to conventional PCR, ELISA and bacterial culture for the diagnosis of paratuberculosis on infected tissues especially from paucibacillary sheep.

## Background

*Mycobacterium avium* subspecies *paratuberculosis* (MAP) is the causative agent of paratuberculosis or Johne’s disease mainly of cattle, goats, sheep and other domestic and wild ruminants. The disease has unusually long incubation period, and is characterised by granulomatous enteritis, diarrhoea, emaciation and mortality. Paratuberculosis occurs throughout the world including India and is responsible for significant economic losses. Animals become infected early in the life but clinical disease develops only in the adult stage Lilenbaum et al. (2007). Thus, animal remains in paucibacillary and subclinical state for a quite long time, excreting none or low numbers of bacilli in the faeces and milk. In these animals, inflammatory responses are quite characteristic to MAP infection, but organisms are difficult to demonstrate Lilenbaum et al. (2007; Perez et al. 1997). Over a period in the infected host, MAP organisms proliferate extensively in tissues which could be easily demonstrable in tissues and faeces by bacterial culture and genetic tests.

Monitoring and surveillance of paratuberculosis at any farm requires an early detection of MAP infection in animals with the aim to further prevent the spread of infection and to reduce the environmental load of MAP. Regular screening of the farm animals by a number of tests including bacterial culture, johnin skin testing, ELISA, PCR and necropsy of the dead animals provide evidence of MAP infection instituting control measures Whittington and Sergeant (2001). Because of the spectral and chronic nature of this disease, none of these tests are 100% sensitive during the entire course of the disease (Ayele et al. 2001; Lilenbaum et al. 2007; Kurade et al. 2004).

At necropsy and histopathology, sheep showing granulomatous enteritis and lymphadenitis with demonstrable and abundant AFB (multibacillary, MB) are tentatively diagnosed as cases of paratuberculosis Clarke and Little (1996). However, in cases where AFB are rare or not demonstrated in granulomatous lesions (paucibacillary, PB), diagnosis is usually difficult and is dependent on tests such as bacterial culture, immunohistochemical methods and conventional PCR or other immunological tests, which have very poor sensitivities. Molecular tests since discovery of IS*900* gene Green et al. (1989), offer a great promise as sensitive and specific diagnostic assays for detection of MAP infection. While conventional PCR-assays provide a rapid means of qualitative assessment, the real-time quantitative PCR (qPCR) assays are endowed with higher sensitivity and help in determining the load of infection in environmental samples, faeces, milk and cultures of MAP (Kawaji, et al. 2007; O'Mahony and Hill 2004). The application of PCR to detect genome directly from tissues is a practical and valuable approach for laboratory confirmation of infectious diseases. The performance of tissue PCR, however, has been less frequently evaluated in sheep for detection of MAP (Clarke and Little 1996; Gwozdz et al. 1997). There have been scant reports on the application and potential of qPCR on tissues of MAP infected sheep and cattle (Smeed et al. 2007; Imirzalioglu et al. 2011).

In this study, we report the efficacy of an IS*900* qPCR in detection and quantification of MAP in sheep tissues with two distinct types of pathology i.e. PB and MB and its comparison with commonly used methods such as bacterial culture, conventional IS*900* PCR and ELISA.

## Materials and methods

### Animals and necropsy

The tissues suspected for Johne’s disease were collected from 56 sheep at the time of slaughter from farms which had history of MAP infection. Detailed history on clinical signs including body weights, progressive emaciation and consistency of faeces were recorded for individual sheep on separate sheet at the time of slaughter. Tissue sections from ileao-caecal junction, proximal and distal ileum and mesenteric lymph nodes (MLN) were collected from each animal. Similarly, tissues (intestines and lymph nodes) from six healthy adult sheep were collected from Municipal slaughterhouse, Bareilly (Uttar Pradesh) and used in the study as control animals. Individual tissues were divided into three parts; one each for PCR and bacterial culture and other for histopathology. All tissues to be used for DNA extraction and bacterial culture were transported to laboratory on ice and stored at −20 °C. For histopathology, tissues were preserved in 10% neutral buffered formalin.

### Histological grading of paratuberculosis lesions

Following fixation, sections of intestines and MLN were trimmed, embedded in paraffin, sectioned at 5 μm and stained with haematoxylin and eosin (HE) for histological examination Culling (1968). The replicate sections from each paraffin block were stained by Ziehl Neelsen’s (ZN) method for the presence of acid-fast mycobacteria. The grading of lesions was performed on the basis of nature and extent of cellular infiltration, location of granuloma in different regions and layers of intestine, and the magnitude of acid-fast bacilli Clarke and Little (1996).

**Figure 1:**
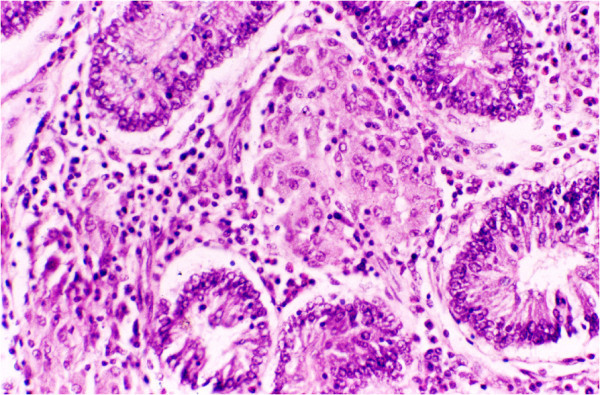
**Small intestine of paucibacillary sheep showing focal granuloma of macrophages surrounded by diffuse infiltration with lymphocytes in basal lamina propria.** H & E.

Paucibacillary sheep did not show any classical clinical symptoms of paratuberculosis. At necropsy, these sheep revealed mild thickening of the terminal ileal mucosa and ileocaecal valve and mild to moderate enlargements of mesenteric lymph nodes. In the ileum and jejunum, villi were club shaped or flattened, and lamina propriae were diffusely infiltrated with lymphocytes and macrophages. Single to multiple small granulomas consisting of large macrophages were observed mostly in the basal lamina propria (Figure 1) and ileal Peyer’s patches. The multinucleated giant cells were also observed in the lamina propria in a number of cases. Mesenteric lymph nodes revealed focal to multiple granulomas and mild macrophage infiltration in the subcapsular, cortical and pericortical areas occasionally with giant cells. Acid-fast bacilli were demonstrated in scarce numbers in 13 cases (86%).

**Figure 2:**
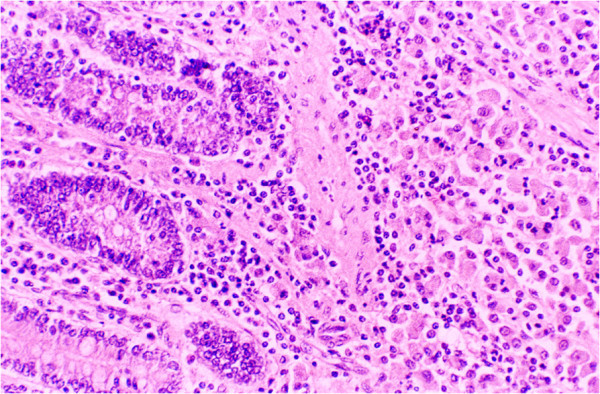
**Diffuse epithelioid cell infiltration in mucosa and submucosa of small intestines in MB sheep.** H & E.

**Figure 3:**
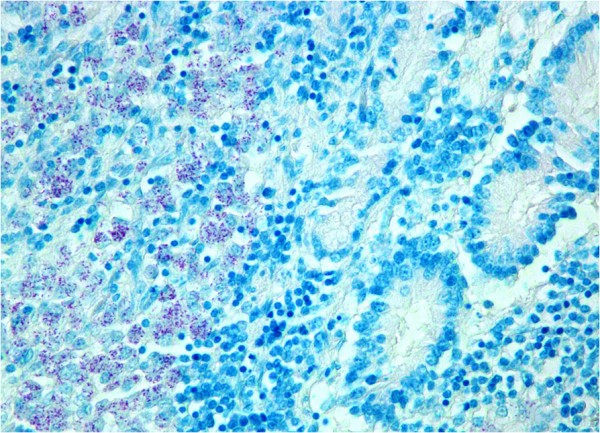
**Small intestinal mucosa and submucosa showing clusters of AFB in the epithelioid cells of multibacillary sheep.** ZN.

Multibacillary group exhibited deterioration in the body conditions, emaciation and moderate to severe loss of body weight, soft faeces and at times diarrhoea. Loss of wool and accumulation of the fluid in the neck regions (bottle jaw condition) was observed in some sheep. At necropsy, gelatinisation of visceral fat, generalized intestinal thickening with prominent corrugations of jejunum and ileum mucosae and enlarged, oedematous, and frequently calcified mesenteric lymph nodes were observed. Predominant histological changes in the small intestines were flat, broad and club shaped villi containing diffuse sheets of acid–fast mycobacteria laden epithelioid cells intermixed with moderate numbers of lymphocytes and polygonal macrophages. In most cases, there was breakdown of muscularis mucosae and the granulomatous lesions extended into the submucosae (Figures 2, 3) and tunica muscularis. The serosal layer in the majority of cases was thickened due to the oedema and loosely aggregated infiltrates of mixed population of lymphocytes and macrophages especially around lymphatics and blood vessels. Acid-fast bacilli were demonstrated in abundance in small intestines and MLN of all multibacillary sheep.

### Culture

Intestinal and lymph node samples from 56 sheep and 6 healthy sheep were processed for bacterial culture separately as described previously Sivakumar et al. (2005). Briefly, 2 g of small intestine or mesenteric lymph node tissues were homogenised in 10 ml sterile distilled water and allowed to settle for 30 min. An amount of 5 ml supernatant was transferred to 50 ml centrifuge tube containing 25 ml of 0.9% hexadecylpyridinium chloride for decontamination. The decontaminated samples were inoculated on Herrold’s egg yolk medium (HEYM, pH 7.4), with and without mycobactin J (Allied Monitor Inc, USA), and incubated at 37°C for 16 weeks. The cultures were examined every 2 weeks for growth and the presence of acid-fast colonies on slopes supplemented with mycobactin J were recorded as positives. All isolates were subsequently confirmed by IS*900* and IS*1311* PCR-REA Marsh et al. (1999).

### Blood samples

Sheep in this study were from farms maintained under semi-intensive system in semi-arid region of Rajasthan. These farms had history of paratuberculosis and was screened with commercial ELISA (Pourquire, France). The procedure was followed as per the instructions of the manufacturer. The S/P ratio lower or equal to 60% was considered to be negative, S/P ratio between 60% and 70% was considered doubtful and S/P ratio equal or greater than 70% was considered positive for MAP infection.

### DNA extraction

Forty four sheep tissues processed for the bacterial culture were used to extract genomic DNA of MAP as per the method described previously with slight modification (Sivakumar et al., 2005; Tripathi et al., 2006). One gram of small intestine and MLN tissues (0.5 g each) was homogenised in 1 ml TE buffer (10 mM Tri, 1 mM EDTA, pH 8.0) in a tissue homogenizer and allowed to settle for 30 min. To 750 μl of supernatant transferred to a fresh sterile microfuge, lysozyme (Sigma, MO, USA) (final concentration 10 mg/ml) was added and incubated at 37°C for 2 h. The suspension was mixed with 10% sodium dodecyl sulphate (SDS) (final concentration 1 mg/ml) and proteinase K (Sigma, MO, USA) (final concentration 2 mg/ml) and incubated at 56°C overnight (16–20 h). The enzyme digested samples were gently mixed with 0.4 volume of 5 M potassium acetate, kept in ice for 10 min and centrifuged at 9600 × g for 10 min. The DNA was purified from the supernatant by adding an equal volume of tris-saturated phenol:chloroform:isoamyl alcohol (25:24:1) (Sigma) and was centrifuged at 9600 × g for 12 min after inverting the tubes for 20 times. The phenol extraction was repeated two to three times. The DNA was precipitated from the aqueous phase by addition of 1/10 volume of cold 3 M sodium acetate and double volumes of absolute ethanol. The resultant DNA pellet was washed with 70% ethanol, dried and resuspended in 50 μl TE buffer.

### Polymerase chain reaction

The primers BA5: 5&’-CTG GCTACC AAA CTC CCG A-3&’, BA6:5&’-GAA CTC AGC GCC CAG GAT-3&’) flanking a region of 314& bp were designed from the IS*900* ( sequence of MAP Bauerfeind et al. (1996). The genomic DNA of MAP isolated from tissues were amplified in 50 μl reaction mixture containing 1X PCR buffer, 2 mM MgCl_2_, 200 mM of dNTPs, 0.5 U of Taq polymerase (MBI Fermantas, MO, USA), 1 μM of primers (BA5 and BA6) and 1 μl of purified genomic DNA solution. The PCR conditions consisted of initial denaturation at 94°C for 4 min, 40 cycles each of denaturation at 94°C for 1 min, annealing at 58°C for 1 min and synthesis at 72°C for 1 min, and final elongation at 72°C for 4 min. In every batch of PCR, positive (DNA from culture of MAP) and negative (DNA from intestine of paratuberculosis negative animals) controls were included. The PCR products were analysed by visualisation of desired size of DNA band in the ethidium bromide-stained agarose gel (1.5%).

### Quantitative real-time PCR (qPCR)

**Figure 4:**
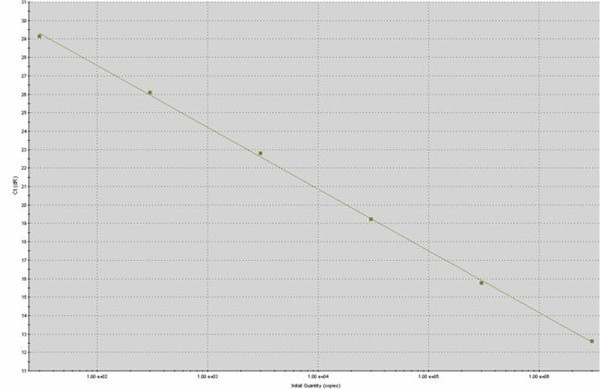
**Standard curve for qPCR assay of MAP IS*900*gene quantification generated by plotting the known DNA concentrations of standard DNA template (3x10**^**7**^**copies) in serial ten-fold dilutions (10**^**-1**^**– 10**^**-6**^**) against the corresponding threshold cycles (Ct values) of the amplification plots with MxPro**^**TM**^**QPCR software.**

The detection as well as the quantification of MAP in small intestinal and MLN tissues of 44 sheep (23 MB, 15 PB and MAP negative control-6) was carried out by a qPCR assay using the same primer set (BA5 and BA6) that generated an amplification product of 314 bp. A quantity of 5 μl tissue DNA (prediluted 1:10) was used as DNA template in all reactions. DNA from a characterized MAP isolate (IVRI-C-132 strain) was used for standardization of qPCR. The qPCR assay was performed with brilliant SYBR green master mix (Stratagene, La Jolla CA, USA) and Mx3000P spectrofluorometric thermal cycler operated by MxPro qPCR software. The PCR conditions included denaturation at 95°C for 10 min (segment I); 40 cycles each consisting of 95°C for 30 s, 58°C for 1 min and 72°C for 30 s (segment II), and the degree of fluorescence was recorded at the end point of each cycle. The dissociation (melting) curve consisting of 95°C for 1 min, followed by 58°C for 30 s, a gradual increase from 58 to 95°C at increments of 2°C per min, and lastly, 95°C for 30 s (segment III) were carried out. After the completion of the dissociation (melting) curve, the melting temperature was recorded as 89.5°C. The copy numbers of the gene of interest per microliter for a known concentration (20 ng/μl) of DNA from a characterized MAP strain (IVRI/C-132) were calculated to construct a standard curve as per the method described previously Yun et al. (2006). The standard curve in terms of a regression line equation was drawn by plotting the known numbers of DNA copies in the dilutions against the threshold cycles (Figure 4). The efficiency of the qPCR assay was found to be 98.8% and RSq value (R2 linear correlation coefficient) was 1.000. Finally, the numbers of copies in the clinical samples were quantified by comparing the amount of MAP DNA against the reference value as per the standard curve. The Ct values falling in between the range of 12.63 to 30.17 with Tm 89.5°C in the test samples were considered positive for MAP genome.

### Statistical analysis

The data obtained from the IS*900* gene PCR, qPCR, bacterial culture and ELISA were analysed by Chi-square test Chandel, (2007).

## Results

### i. Pathology

Out of 56, 38 sheep were found to have histological lesions characteristic to paratuberculosis. Eighteen sheep had other lesions/conditions such as acute/subacute enteritis, parasitic infestation, hepatitis, suppurative nodules etc. All 38 sheep, were classified in two groups, “paucibacillary” (n = 15) and “multibacillary” (n = 23).

### ii. ELISA

Out of blood samples from 27 sheep (8 PB and 19 MB) available for testing, 21 (77.7%) were found to be positive for antibody to MAP. The positive sheep included 8 (25%) PB and all 19 (100%) MB cases (Table 1). The six cases negative in the ELISA has S/P ratio between −34.52 to 26.17.

**Table 1:** **Comparative efficacy of bacterial culture, ELISA, conventional and qPCR of IS*900*gene in PB and MB sheep tissues**

Pathology	Bacterial culture	ELISA	IS900 PCR positive	qPCR positive
Multibacillary	14/23 (60.9%)^Aa^	19/19 (100%)^Aa^	19/23 (82.6%)^Aa^	23/23 (100%)^Ab^
Paucibacillary	05/15 (33.3%)^Bab^	2/8 (25%)^Ba^	02/15 (13.3%)^Bb^	15/15 (100%)^Ac^
**Total sheep tested**	**19/38 (50%)**^a^	**21/27 (77.7%)**^a^	**21/38 (55.2%)**^a^	**38/38 (100%)**^b^

Values bearing different superscripts within a column (capital letter) and row (small letter) differ significantly at 5% level (P < 0.5).

### iii. Bacterial culture

Healthy control sheep and eighteen sheep which were devoid of granulomatous lesions, did not yield MAP culture. Tissues of 14 MB and 5 PB sheep, either intestines or lymph nodes were found to be positive for MAP on the basis of their colony morphology, slow growth, mycobactin dependency and IS*900* PCR. In three MB sheep mycobacterial colonies were visible after 4 weeks of inoculation. These colonies were white opaque and translucent. Similar colonies in 10–12 numbers were observed after 10 weeks in 11 MB sheep. In 5 PB sheep, the colony growths were very slow and 4–8 small white colonies were visible after 17 weeks of inoculation. All isolates were found to be positive in IS*900* PCR and were identified as “bison” (B) type strains.

### iv. Conventional PCR

**Figure 5:**
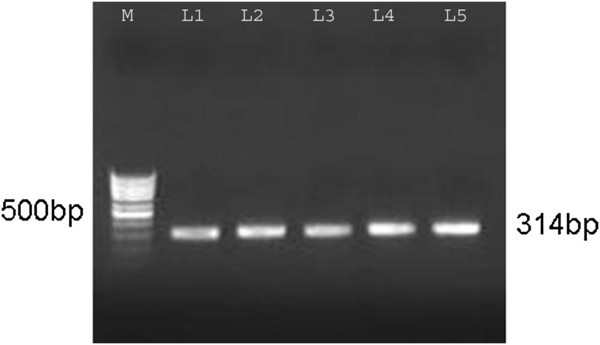
**IS900 gene PCR for the detection of MAP in intestinal and MLN tissues of naturally MAP infected sheep.** Lane M-Marker, L 1- Positive control, L 2- AV22 (Int.), L 3 – AV23 (MLN), L4 – AV31(Int.), L5 – AV31(MLN).

IS*900* PCR detected 21 (55.2%) sheep positive, of which 19 (82.6%) were multibacillary and 2 (13.3%) were paucibacillary cases (Table 1; Figure 5). There was no significant difference in the detection rate of MAP genome between the small intestine and mesenteric lymph node tissues.

### v. qPCR

**Figure 6:**
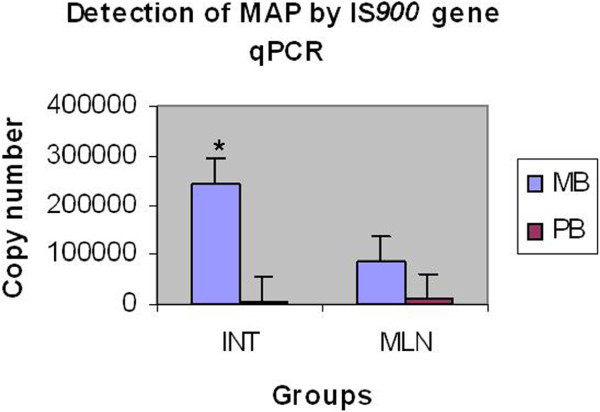
**Detection of MAP by IS*900*gene qPCR in intestinal and MLN tissues.**

The qPCR results in terms of copy numbers obtained for the tissue samples of intestine and lymph nodes have been presented in Tables 2 and 3. Among 23 MB sheep, the assay detected an average of 2.4 × 10^5^ copies (range 1.040 × 10^3^ to 1.092 x 10^6^) in the small intestines and 8.5 × 10^4^ copies (range 1.55 x 10^3^ to 7.729 × 10^5^) in the mesenteric lymph nodes. Among 15 PB sheep, average detection was 7.2 × 10^3^ (range 9.083 × 10^2^ to 1.927 × 10^4^) in small intestine and 1.1 × 10^4^ copies in MLN (range 8.154 × 10^2^ to 1.073 × 10^5^). It is evident from these results that MAP genome contents were higher in the intestines as compared with the MLN in both PB (23 times) and MB (3 times) sheep. A further analysis between groups (PB vs MB) revealed that intestines and MLN of MB sheep contained 34 and 23 times greater MAP genome in comparison to corresponding tissues of PB sheep, respectively (Figure 6).

**Table 2:** **Results of culture, ELISA, ZN, IS900 PCR and qPCR in MB tissues**

Sr.No	Animal Id	Pathological Type	Culture	ELISA (SP)	ZN	IS900 PCR	qPCR	
							Int.	MLN
1	AV1	Multibacillary	+	232.73	+	+	1.399 × 10^4^	5.008 × 10^4^
2	AV2	Multibacillary	+	95.57	+	+	2.274 × 10^4^	2.738 × 10^4^
3	AV3	Multibacillary	+	151.54	+	+	9.09 × 10^4^	5.653 × 10^3^
4	AV9	Multibacillary	-	115.2	+	+	1.85 × 10^4^	1.77 × 10^3^
5	AV10	Multibacillary	-	128.12	+	+	9.69 × 10^3^	1.553 × 10^3^
6	AV11	Multibacillary	+	119.24	+	-	1.26 × 10^5^	2.558 × 10^3^
7	AV12	Multibacillary	-	135.24	+	+	3.42 × 10^4^	1.974 × 10^3^
8	AV13	Multibacillary	-	176.73	+	+	4.482 × 10^4^	1.994 × 10^3^
9	AV14	Multibacillary	+	135.24	+	+	5.53 × 10^4^	6.014 × 10^3^
10	AV22	Multibacillary	+	103.81	+	+	7.056 × 10^5^	1.491 × 10^5^
11	AV23	Multibacillary	+	160.41	+	+	8.536 × 10^5^	9.758 × 10^4^
12	AV25	Multibacillary	+	120.11	+	+	2.276 × 10^4^	1.411 × 10^5^
13	AV26	Multibacillary	-	139.56	+	+	1.092 × 10^6^	4.682 × 10^3^
14	AV28	Multibacillary	+	120.31	+	+	1.383 × 10^5^	1.771 × 10^4^
15	AV29	Multibacillary	-	80.08	+	+	6.265 × 10^5^	1.285 × 10^5^
16	AV31	Multibacillary	+	131.55	+	+	8.765 × 10^4^	5.009 × 10^3^
17	AV32	Multibacillary	+	84.62	+	+	7.193 × 10^5^	1.137 × 10^5^
18	AV34	Multibacillary	+	90.79	+	+	4.043 × 10^4^	4.590 × 10^4^
19	AV36	Multibacillary	+	70.42	+	+	6.313 × 10^5^	3.278 × 10^5^
20	AV42	Multibacillary	-	NT	+	-	7.122 × 10^4^	7.729 × 10^5^
21	AV43	Multibacillary	-	NT	+	-	1.040 × 10^3^	8.026 × 10^3^
22	AV45	Multibacillary	-	NT	+	-	2.31 × 10^3^	1.859 × 10^3^
23	AV52	Multibacillary	+	NT	+	+	2.059 × 10^5^	3.876 × 10^4^

*Int* = Intestine, *MLN* = Mesenteric lymph node, *NT* = Not tested, + = Positive, - = Negative, SP > 70 is positive.

**Table 3:** **Results of culture, ELISA, ZN, IS900 PCR and qPCR in PB tissues**

Sr.No	Animal Id	Pathological Type	Culture	ELISA (SP)	ZN	IS900 PCR	qPCR	
							Int.	MLN
1	AV24	Paucibacillary	+	3.29	+	-	1.582 × 10^4^	1.073 × 10^5^
2	AV27	Paucibacillary	+	2.71	+	-	1.775 × 10^4^	1.046 × 10^4^
3	AV30	Paucibacillary	-	1.04	+	-	6.141 × 10^3^	1.565 × 10^3^
4	AV33	Paucibacillary	+	3.715	+	-	1.296 × 10^4^	6.637 × 10^3^
5	AV35	Paucibacillary	-	86.11	+	-	8.441 × 10^3^	4.209 × 10^3^
6	AV37	Paucibacillary	+	136.33	+	-	9.991 × 10^3^	3.404 × 10^3^
7	AV38	Paucibacillary	-	18.75	-	-	3.369 × 10^3^	1.734 × 10^3^
8	AV39	Paucibacillary	-	26.17	-	+	3.501 × 10^3^	2.904 × 10^3^
9	AV41	Paucibacillary	-	NT	+	-	1.292 × 10^3^	8.279 × 10^2^
10	AV44	Paucibacillary	-	NT	+	-	1.123 × 10^3^	9.158 × 10^2^
11	AV46	Paucibacillary	-	NT	+	+	9.083 × 10^2^	8.154 × 10^2^
12	AV48	Paucibacillary	+	NT	+	-	1.054 × 10^3^	1.369 × 10^3^
13	AV49	Paucibacillary	-	NT	+	-	1.927 × 10^4^	7.95 × 10^3^
14	AV50	Paucibacillary	-	NT	+	-	1.238 × 10^3^	8.640 × 10^3^
15	AV51	Paucibacillary	-	NT	+	-	4.935 × 10^3^	3.08 × 10^3^
16	AV67	Uninfected control	-	5.312	-	-	-	-
17	AV68	Uninfected control	-	−21.23	-	-	-	-
18	AV69	Uninfected control	-	1.112	-	-	-	-
19	AV70	Uninfected control	-	13.653	-	-	-	-
20	AV71	Uninfected control	-	−23.114	-	-	-	-
21	AV72	Uninfected control	-	−34.52	-	-	-	-

*Int* = Intestine, *MLN* = Mesenteric lymph node, *NT* = Not tested, *+* = Positive, - = Negative, SP > 70 is positive.

## Discussion

Due to insidious nature of MAP infection in animals, prolonged incubation period and detection of disease only in adult animals, the control and eradication of Johne’s disease has been difficult throughout the world. In early stages of the infection, where excretion of the bacilli is too low to be detected by culture or PCR, regular screening of morbid tissue materials in histopathology and subsequent confirmation by culture or genetic test appear to be a logical option for detecting incursion of MAP infection in the farm. Since ELISA as a clinical test is most commonly used in the screening of animals, we wanted to see that how it performs in relation to pathology and other tests conducted on tissues.

The sensitivity of IS*900* PCR observed in MB sheep (82.6%) in the present study was found to be in accordance with those reported previously in sheep and goats (90-100%) (Clarke and Little 1996; Gwozdz et al. 1997; Tripathi et al. 2006). Sensitivity of IS*900* PCR (13.3%) observed in PB sheep of the current study was lower in comparison to previous reports of 66% in PB goats Tripathi et al. (2006) and 50% in mildly infected experimental sheep Anand Kumar (2004). Clarke and Little (1996) also reported lower sensitivity in PB (64%) cases as compared to MB (100%) sheep. The negative PCR results in PB cases could be due to low number of bacteria present in tissues, i.e. below the detection limit (230 bacilli/g) of the procedure used in the study (Clarke and Little 1996; Sivakumar et al. 2005; Tripathi et al. 2006).

The qPCR assay in the present study was found to be 100% sensitive in the detection of MAP genome in both forms of the disease. Imirzalioglu et al. (2011) detected MAP DNA in all 9 gut inflamed tissues from cattle using multiple qPCR targeting IS*900*, 251 locus, F57 and MAP0865 genes. None non-inflammed tissues from 3 healthy cattle were positive in multiple qPCR. The higher sensitivity of real-time PCR was also reported previously in spiked milk O'Mahony and Hill (2004), in the cattle faeces (Irenge, et al. 2009; Kawaji, et al. 2007), in the artificially infected bovine semen Herthnek et al. (2006) and in the laboratory cultures of MAP Rajeev et al. (2005). Sweeney et al. (2005) reported 100% sensitivity of the qPCR in heavy and moderate shedders, and 30% sensitivity in light shedders in bovine faecal samples. In a recent study, qPCR assay on faecal samples detected 8 of 13 (61.5%) experimentally MAP inoculated sheep, 14 of 208 (6.7%) sheep kept on pasture with a low level of MAP contamination, 24 of 40 (60%) faecal culture negative but histologically and/or tissue culture positive sheep and 68 of 69 (98.6%) culture positive sheep. Overall, qPCR has been reported to be more sensitive (76.5%) than faecal culture (41.2%) in the detection of MAP genome in faeces (Kawaji et al., 2007). In our study, MAP DNA (9.083 × 10^2^ to 1.927 × 10^4^ copies / 5 μl) was detected by qPCR in 10 culture negative tissues of PB sheep which had occasional/sparse AFB on ZN stained tissue section examination. Eight of these 10 PB sheep was also negative in IS*900* PCR. Thus, our results suggest higher sensitivity of the qPCR assay carried out in the present study.

When the quantity of MAP genome was compared in the intestine and MLN of the PB and MB sheep, the MAP genome copies in MB intestine were found to be significantly higher than MLN (about 3 times) of the MB sheep and intestine (about 34 times) and MLN (about 23 times) of the PB sheep. In a previous study, it was reported that mycobacteria were numerous in intestinal mucosa, relatively less numerous in the submucosa, regional lymph nodes and in other organs of MB sheep without any quantitative estimation Clarke and Little (1996). This difference was probably due to the fact that lymph nodes have better resistance in the form of activated CD4+ cells and IL-2 cytokines to control the proliferations of MAP than those of intestines, where lymphoid tissues are discretely present in the form of Peyer’s patches in adult sheep (Clarke and Little 1996; Valheim et al. 2002; Coussens et al. 2004).

We consider that the difference of MAP genomic content between PB and MB sheep on qPCR analysis though significant does not appear to be huge as it is reflected in ZN stained tissue sections. While in MB sheep, AFB were abundant and detectable even at lower magnification, in PB sheep, there were only few/occasional bacilli and detectable only with oil immersion microscopy. The possible explanation could be the presence of MAP in PB tissues in spheroplast forms in the host immunological niche Clarke (1997), degraded forms or their presence may not be associated with granuloma as suggested in ruminants and Crohn’s disease (CD) patients (Behr and Kapur 2008; Jeyanathan et al. 2007; Perez et al. 1996).

In the present study, bacterial culture detected 50% sheep positive, which were in agreement with previous studies reporting 16 to 71% sensitivities on tissues (Clarke and Little 1996; Perez et al. 1996). Better sensitivity obtained in MB sheep (60.9%) over PB sheep (33.3%) was in line with previous reports suggesting that mycobacterial load in the tissues was positively correlated with the culture sensitivity Perez et al. (1996). Non-isolation of MAP from tissues of several MB and PB cases may be due to several reasons. It has been shown that MAP has a state of dormancy and may not always be cultured even though the organisms are viable Whittington et al. (2004). Bacterial culture is influenced by contamination or overgrowth of other gut flora and/or potential reduction in the number of viable MAP by several steps of decontamination procedures. It may also be attributed to the existing limitations of conventional culture method, especially in small ruminants (Sigurdardottir et al. 1999; Whittington and Sergeant 2001; Kurade et al. 2004). A previous study reporting improved sensitivity by simultaneous culture on HEY and LJ media Aduriz et al. (1995) suggested use of both media to obtain better sensitivity of the bacterial culture in sheep.

ELISA detected 77.2% of all cases with 100% detection rate in MB and 25% in PB sheep, which were in agreement with previous studies in sheep and goats where 10-87% sensitivities were reported (Clarke et al. 1996; Perez et al. 1997; Tripathi et al. 2006). Another study reported 48% of 62 paratuberculous sheep to be positive in absorbed ELISA and advocated that a close relationship existed between pathologic findings and the serological response Perez et al. (1997). If results of all the tests are compared in MB cases, qPCR and ELISA were 100% sensitive followed by PCR (82.6%) and bacterial culture (60.9%), thus showing the importance of ELISA in clinical cases. However, qPCR was found to far more sensitive (100%) than culture, PCR and ELISA (13.3 to 33.3%) in PB sheep.

## Conclusion

Results of the present study indicated the utility of qPCR assay in rapid, sensitive and specific detection of MAP in the tissues of naturally infected sheep in comparison to conventional PCR, bacterial culture and ELISA. The higher sensitivity of the qPCR assay especially in paucibacillary sheep underlines its value in the retrospective diagnosis and control of the disease. The ELISA results of the present study detecting all multibacillary sheep highlight its value in the diagnosis of clinical cases.

## Authors’ information

GG is a senior scientist and works on molecular pathogenesis and diagnosis of paratuberculosis and other infectious diseases. BN is currently Director of CCS National Institute of Animal Health, Baghpat (UP) and works on immunopathobiology and diagnosis of bacterial and viral diseases with special interest on paratuberculosis research.
